# Harnessing preclinical simulation to build capacity in physiotherapy education and practice

**DOI:** 10.4102/sajp.v82i2.2308

**Published:** 2026-04-30

**Authors:** Ronel Roos, Sandy Lord, Monique Keller, Nabeela Sujee, Natalie Benjamin-Damons, Vaneshveri Naidoo

**Affiliations:** 1 Department of Physiotherapy, Faculty of Health Sciences, University of the Witwatersrand, Johannesburg, South Africa; 2 Centre for Health Science Education, Faculty of Health Sciences, University of the Witwatersrand, Johannesburg, South Africa

## Context

The transition from preclinical to clinical training is an important part of the journey of a physiotherapy student. Transitioning to clinical practice involves overlapping shifts in knowledge, identity and the environment, often accompanied by anxiety as students begin working with unfamiliar patients in new environments (Jindal-Snape 2023). According to Stoikov et al. (2022), physiotherapy students report a lack of preparedness to practice because of limited exposure to diverse and complex conditions and high-performance expectations, which place additional stress on them. Integrating early clinical exposure into the preclinical curriculum can enhance the relevance of theoretical knowledge and potentially improve learning outcomes.

Experiential learning not only promotes technical competence but also helps form students’ professional identity and nurtures emotional, cognitive and interpersonal growth (Korpi, Piirainen & Peltokallio 2017). In the foundational years of the undergraduate physiotherapy curriculum, students may have limited clinical exposure, with theory and practical content delivered in a blended teaching format. Practical sessions are included, where students often practice the clinical skills they have learned on one another. Clinical education, during these formative years, may consist of students observing physiotherapy practice by shadowing a qualified physiotherapist or senior student during work-based learning on the clinical platform. These initial encounters, while valuable, lack depth of interaction, particularly the lived experience of engaging, handling and managing an unknown individual (patient) in a clinical setting.

Simulation-based education (SBE) offers a means of bridging the gap between theory and practice. The perspective discussed in our scientific letter explores the pivotal role of SBE and establishing a physiotherapy community of practice (CoP) during a preclinical simulation experience as key strategies for enhancing physiotherapy education and practice.

## Simulation-based education – Theory and practice

Simulation-based education is an innovative pedagogy designed to enhance teaching and learning in health professions education. It replicates realistic clinical scenarios in a safe environment, allowing students to demonstrate clinical reasoning and skills without risk to real patients. Structured debriefing that supports reflective learning follows these experiences.

Kolb’s Experiential Learning Theory, Constructivism and Reflective Practice underpin SBE (Squires et al. 2024). Kolb’s theory suggests that learning and knowledge creation occur through a cyclical process involving *concrete experience* (feeling), *reflective observation* (reviewing), *abstract conceptualisation* (thinking) and *active experimentation* (doing). In simulation, students engage in authentic clinical scenarios that emulate clinical practice (*concrete experience*), which allows them to execute clinical skills (*active experimentation*) through clinical reasoning and clinical decision-making (*abstract conceptualisation*). *Reflective Observation* is facilitated through Schön’s reflection-in-action during simulation, and reflection-on-action, by means of structured debriefing at the conclusion of the simulation. Reflection is pivotal in simulation promoting deep learning and professional development (Aitken et al. 2021). Constructivism (social and cognitive) complements experiential learning as learners construct knowledge through active engagement, collaboration and situated and contextualised learning (Squires et al. 2024).

Simulations may involve manikins or simulated patients (SPs). Simulated patients are individuals trained to portray patients authentically. Simulated patients provide clinical histories and embody the behaviours and mannerisms associated with specific conditions, adding realism and emotional depth to the encounter (Cleland, Abe & Rethans 2009). This immersive experience enables students to engage meaningfully, fostering professional growth and the development of clinical identity. Debriefing offers students a space to reflect not only on clinical decisions but also on their emotional responses and interpersonal interactions. Effective debriefing uses structured approaches and is guided by the principle of ‘good judgement’, where facilitators adopt a stance of curiosity (Rudolph et al. 2007). This approach helps to uncover students’ frames of reference, encouraging deeper reflection and the identification of strategies for improved practice.

Peer assisted teaching and learning (PATL) within SBE contexts further enhance the educational experience by enabling students to take on both learner and educator roles, promoting deeper understanding and reinforcing knowledge retention (Aljahany et al. 2021). In addition, the use of students as SPs offers a unique dimension to SBE, providing authentic interactions and feedback while simultaneously benefiting both the ‘patient’ and the practicing student (Dalwood et al. 2020). This multifaceted approach to SBE not only prepares students for real-world clinical encounters but also cultivates essential skills such as communication, empathy and professionalism, ultimately contributing to improved patient care and safety in healthcare settings.

## The preclinical simulation experience

A preclinical simulation experience, consisting of multiple cases with SPs, is used to provide second-year physiotherapy students with clinical exposure at the University of the Witwatersrand. The SPs are postgraduate physiotherapy students, assigned to a clinical case as per their area of clinical expertise. The SPs are trained on the objectives of the session, their role during the simulation, the knowledge base of the second-year students and the dos and don’ts when simulating a patient. Information on the clinical cases and the objectives of this SBE are published to the undergraduate students well in advance to allow adequate time for preparation. The cases range from a patient in a medical ward presenting with hemiplegia visited by a caregiver to a patient attending an outpatient clinic complaining of a painful shoulder.

Learning objectives focus on optimising students’ communication skills, providing the students with an opportunity to conduct and implement patient assessment and treatment skills across physiotherapy specialities on a SP, and to do a self-reflection on practice. In addition, participating as an ‘educator’, observing and providing feedback to peers on their patient management following the simulation.

**FIGURE 1:**
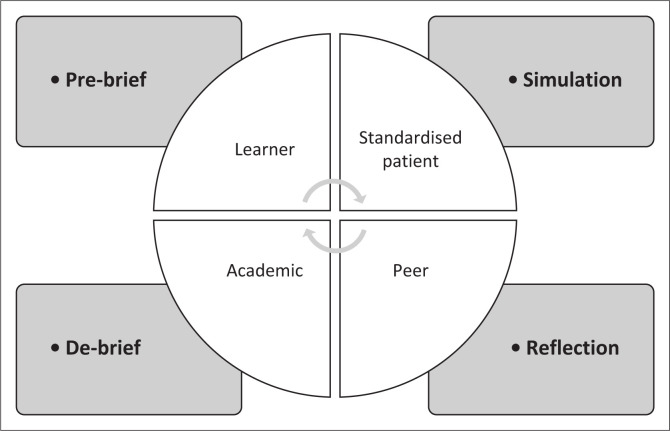
Preclinical simulation within the physiotherapy community of practice.

On the simulation day, students receive a prebrief from an academic who serves as a facilitator and their peers, as per their allocated simulation group. Queries are clarified, and brainstorming with peers regarding their assigned case is encouraged. The simulation with the SPs then follows, during which a peer observes the patient management. Following the simulation, a debriefing at the bedside of the SP occurs, consisting of reflection, feedback and discussions with the postgraduate student, peer and the facilitating academic regarding their case management (Figure 1). Then, a general debriefing, with another academic follows concerning the SBE as a whole. Students provide feedback on this SBE experience using a standardised tool.

## A community of practice in action

A CoP, coined by Lave and Wenger (1991), emphasises the social nature of how learning occurs in a community of professionals with a pre-set core knowledge, skills, values and norms upheld by a professional community as a shared identity. A CoP, therefore, can be defined as an ongoing, sustained network of individuals at undergraduate, postgraduate or continued professional development levels who share overarching knowledge bases, values, history, beliefs and shared practice experiences. Cruess, Cruess and Steinert (2018) emphasise that a CoP can provide a theoretical basis for a variety of educational activities, especially in health sciences education, which takes place within the profession’s community. A CoP therefore assists students (undergraduate and postgraduate) as well as educators navigate the complex nature of the respective healthcare contexts within which they function.

In the preclinical simulated clinical practice environment, the community of physiotherapy practitioners, namely, the second-year students, postgraduate students acting as SPs, and academics, assist the novice CoP members, through pre-briefing, assisted reflection, feedback and debriefing. The three essential CoP elements, namely, domain, community and practice, facilitate the acquisition of knowledge, skills and professional behaviours of working with patients in a clinical setting to support the undergraduate students’ transition from peripheral participation to full membership as future colleagues (Cruess et al. 2018).

## Essential elements for consideration

Considering the aforementioned in this scientific letter, the following are essential elements for deliberation when implementing a preclinical simulation experience, including multiple cases:

- Developing, organising, implementing and maintaining annual multicase simulations is a resource intensive endeavour. Without committed and enthusiastic staff who are dedicated to teaching, learning and the advancement of clinical education, successful execution is not achievable.
- It is vital that SPs reflect authentic behaviours, refrain from acting the outlier, ‘difficult patient’, and rather just be the ‘normal’ patient. This is especially important within the context of the preclinical student body with limited clinical exposure experience.
- Having postgraduate students, who are physiotherapists, with the clinical background of the patient that he or she represents, is immensely beneficial in creating a realistic clinical encounter. Additionally, having them facilitate the bedside debriefing, feedback and discussions with the undergraduate students is a valuable resource.
- Teamwork is fundamental to foster an environment that promotes innovation in SBE and clinical education.

## Conclusion

Peer-assisted teaching and learning, in the form of the established physiotherapy CoP (second-year students, postgraduate students and academics), enhances learning by promoting critical reflection, reinforcing clinical reasoning and strengthening professional identity across all levels of the physiotherapy community. Simulation-based education, embedded within this CoP, provides a structured and collaborative framework for supporting physiotherapy students’ clinical education and strengthening physiotherapy education and practice.
